# The Combined Effect of Environmental and Host Factors on the Emergence of Viral RNA Recombinants

**DOI:** 10.1371/journal.ppat.1001156

**Published:** 2010-10-21

**Authors:** Hannah M. Jaag, Peter D. Nagy

**Affiliations:** Department of Plant Pathology, University of Kentucky, Plant Science Building, Lexington, Kentucky, United States of America; Centro de Biología Molecular Severo Ochoa (CSIC-UAM), Spain

## Abstract

Viruses are masters of evolution due to high frequency mutations and genetic recombination. In spite of the significance of viral RNA recombination that promotes the emergence of drug-resistant virus strains, the role of host and environmental factors in RNA recombination is poorly understood. Here we report that the host Met22p/Hal2p bisphosphate-3′-nucleotidase regulates the frequency of viral RNA recombination and the efficiency of viral replication. Based on *Tomato bushy stunt virus* (TBSV) and yeast as a model host, we demonstrate that deletion of *MET22* in yeast or knockdown of *AHL*, *SAL1* and *FRY1* nucleotidases/phosphatases in plants leads to increased TBSV recombination and replication. Using a cell-free TBSV recombination/replication assay, we show that the substrate of the above nucleotidases, namely 3′-phosphoadenosine-5′-phosphate pAp, inhibits the activity of the Xrn1p 5′-3′ ribonuclease, a known suppressor of TBSV recombination. Inhibition of the activity of the nucleotidases by LiCl and NaCl also leads to increased TBSV recombination, demonstrating that environmental factors could also affect viral RNA recombination. Thus, host factors in combination with environmental factors likely affect virus evolution and adaptation.

## Author Summary

Viral RNA recombination plays a major role in virus evolution. Yet, we know little about the roles of host and environmental factors in viral RNA recombination. In this work, using TBSV and yeast as a model host, we show that *MET22* nucleotidase suppresses viral RNA recombination. In vitro experiments with a cell-free extract from yeast revealed that the substrate of Met22p bisphosphate-3′-nucleotidase, namely pAp, could promote TBSV RNA recombination via inhibiting the activity of the Xrn1p 5′-3′ ribonuclease, a known suppressor of viral RNA recombination. Altogether, we demonstrate that the *Met22/Xrn1* pathway and environmental factors affecting this pathway, namely salt-stress caused by LiCl and NaCl, play a role in viral RNA recombination. The authors also provide evidence that a similar pathway affects TBSV recombination in plants as well. The most pronounced increase in accumulation of TBSV RNA recombinants was seen in the triple-nucleotidase gene silenced plants treated with LiCl, suggesting that the combined effect of genetic and environmental factors could be critical in regulation of the rate of viral RNA recombination.

## Introduction

Viruses with RNA genomes are abundant pathogens of plants and animals. Many RNA viruses have ultrafast replication cycles and rapid evolution, leading to the continuous emergence of new strains and variants. In addition to high frequency mutations and genome reassortments for multicomponent RNA viruses [1], [2], [3], [4], RNA recombination is one of the major driving forces in RNA virus evolution, helping viruses to invade new hosts, develop resistance against drugs and other antivirals and form more virulent strains [3], [5], [6], [7]. Another benefit of RNA recombination is the increased fitness of viruses in some hosts [8]. However, natural selection pressure on the recombinant viruses could be a significant limiting force during their spread, keeping recombinant and parent viruses close to an “evolutionary optimum” level.

RNA recombination also functions in the repair of viral RNA molecules by utilizing truncated/damaged viral RNA molecules [9], [10], [11], [12]. The repair function of RNA recombination might compensate viruses for the high mutation rate, which could introduce detrimental mutations into the viral genomes, reducing the fitness of clonal viral populations [3], [13]. Thus, RNA recombination can also be regarded as a guardian of the viral genome, and its second function is to increase genome variability.

Viral RNA recombination leads to the joining of two or more noncontiguous segments of the same RNA or two separate RNAs together [14]. Recombination is thought to be a frequent event during the infectious cycles of some RNA viruses [5], [6]. Most RNA recombination is based on template switching by the viral polymerase, as documented by in vitro approaches for a number of viruses [15], [16], [17], [18], [19], [20]. In spite of the high frequency RNA recombination for some viruses, the detection of recombinant viral RNAs could be challenging since most of the recombinants are likely poorly adapted to their environment and therefore recombinant viral RNAs are eliminated rapidly from viral populations. Comparison of viral RNA genomes, however, reveals that recombination has shaped the evolution of many RNA viruses [6]. Studies on the viral replication proteins have revealed their roles in RNA recombination events and led to template-switching recombination model as the most widespread mechanism during recombination events [14], [21], [22]. Moreover, sequences/structures in the viral RNA could act as hot- or cold-spots in promoting or inhibiting viral RNA recombination, respectively [14], [23], [24], [25], [26]. Altogether, RNA recombination seems to be a dynamic and probabilistic event that shapes the population of viruses by contributing to virus variability, but also serving as a genome repair mechanism to maintain the infectivity of RNA viruses [3], [14].

In spite of our increasing knowledge about viral RNA recombination over the last two decades that contributed new insights into the roles of viral proteins and the viral RNA in RNA recombination [14], the roles of host proteins and environmental factors are poorly understood. Genome-wide screens of ∼5,500 yeast knock out and knock down strains and proteomics approaches with 4,100 purified yeast proteins involving *Tomato bushy stunt virus* (TBSV), a tombusvirus infecting a wide range of plants, and yeast model host have revealed that several dozens of host genes could affect viral RNA recombination either directly or indirectly [27], [28], [29], [30], [31]. One of the most critical host factors identified is the cytosolic Xrn1p 5′-3′exoribonuclease (Xrn4 in plants). This exoribonuclease can reduce the frequency of TBSV recombination by efficiently degrading RNA recombination intermediates [32], [33], [34]. Other ribonucleases, which are components of the RNA silencing pathway, also affect recombination of a fungal RNA virus, suggesting that ribonucleases might affect the evolution of a range of RNA viruses [35], [36].

In this work, we tested the role of the previously identified *MET22*/*HAL2* gene, whose deletion increased TBSV RNA recombination in yeast [30]. *MET22* codes for a bisphosphate-3′-nucleotidase in the sulfate assimilation pathway involved in methionine biosynthesis and it affects salt tolerance [37]. Met22p removes the 3′ phosphate from 3′-phosphoadenosine-5′-phosphate (pAp), thus producing AMP, as well as hydrolyzing 3′-phosphoadenosine 5′-phosphosulfate (pApS) [38], [39] and is also active on other biphosphorylated nucleotides (pNp) [40]. Our working model was that deletion of *MET22* might promote TBSV RNA recombination by leading to increased cytosolic level of pAp [41] and subsequent pAp-mediated inhibition of the ribonuclease activity of Xrn1p in yeast cells [37]. The reduced Xrn1p activity would then lead to increased TBSV recombination due to the elevated levels of short RNA recombination intermediates that are not degraded by Xrn1p efficiently in the presence of pAp [32], [33], [34].

To test this model, we complemented *met22*Δ yeast with Met22p mutants defective in bisphosphate-3′-nucleotidase function, which did not suppress TBSV recombinant RNA accumulation, suggesting that the enzymatic function of Met22p is important to inhibit TBSV recombination. In addition, inhibition of Xrn1p exoribonuclease via pAp in a cell-free TBSV replication assay demonstrated increased accumulation of TBSV recombination products as well as enhanced level of partial degradation products of TBSV replicon (rep)RNA, which are intermediates in RNA recombination [34]. Inhibition of the Met22p activity with either LiCl or NaCl also increased TBSV recombination, suggesting that environmental factors, such as salt stress, could affect viral RNA recombination.

## Results

### Met22p nucleotidase affects the accumulation of TBSV recombinant RNAs in yeast cells

**Figure 1:**
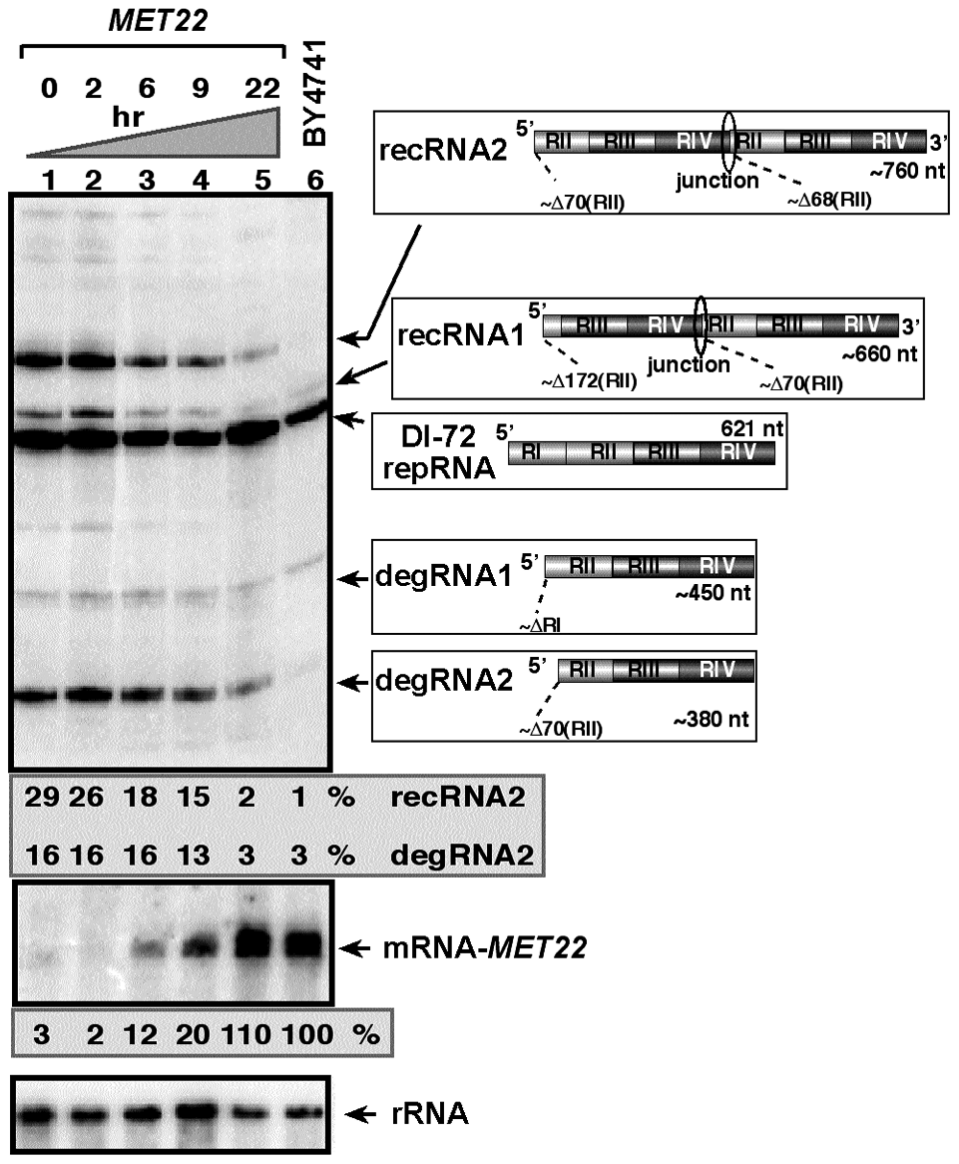
Expression of Met22p bisphosphate-3′-nucleotidase inhibits TBSV RNA recombination in yeast. Top image: Northern blot analysis for detection of (+)-strands of TBSV DI-72 repRNA and recRNAs from wt and *Gals-met22* yeast strains. The wt Met22p was expressed from the *GALS* promoter in *Gals-met22* strain for the time shown on the top of the panel. The accumulating repRNA and the newly formed recRNAs are shown with arrows. The numbers at the bottom of the panel show the percent of recRNA and degRNA2 accumulation for the *Gals-met22* yeast, which is normalized to the level of DI-72 repRNA (100% in each sample). The various 5′-to-3′ sequences present in the repRNA and the generated recRNAs and degRNAs are shown on the right. The two major groups of recRNAs, recRNA1 and recRNA2, are shown schematically. The middle and bottom images show the Northern blot analyses of *MET22* mRNA and ribosomal RNA levels, respectively, in the corresponding samples.

To confirm that Met22p affects TBSV recombinant (rec)RNA accumulation, we expressed Met22p from the weak galactose-regulatable *GALS* promoter from its original chromosomal location in BY4741 yeast (*Gals-met22*) that also carried the plasmids for launching TBSV repRNA accumulation [42], [43]. Culturing yeast for 22 hours in a media containing galactose led to suppression of recRNA accumulation by ∼15-fold when compared to *Gals-met22* yeast cultured in a media containing glucose that represses the *GALS* promoter (compare lanes 1 and 5, Fig. 1) [44]. Interestingly, the accumulation of partly degraded TBSV repRNAs, named degRNAs [32], [34], was also suppressed by ∼5-fold in *Gals-met22* yeast grown for 22 hr in the presence of galactose. These degRNAs represent 5′-truncated TBSV repRNAs (shown schematically in Fig. 1) [30], [34]. The accumulation levels of recRNAs and degRNAs in *Gals-met22* yeast grown for 22 hr in the presence of galactose were only a little bit higher than the levels of recRNAs and degRNAs observed in the wt BY4741 yeast expressing Met22p from its original promoter (compare lanes 5 and 6, Fig. 1), suggesting that *MET22* is responsible for affecting the generation and accumulation of TBSV recRNAs and degRNAs in yeast cells.

**Figure 2:**
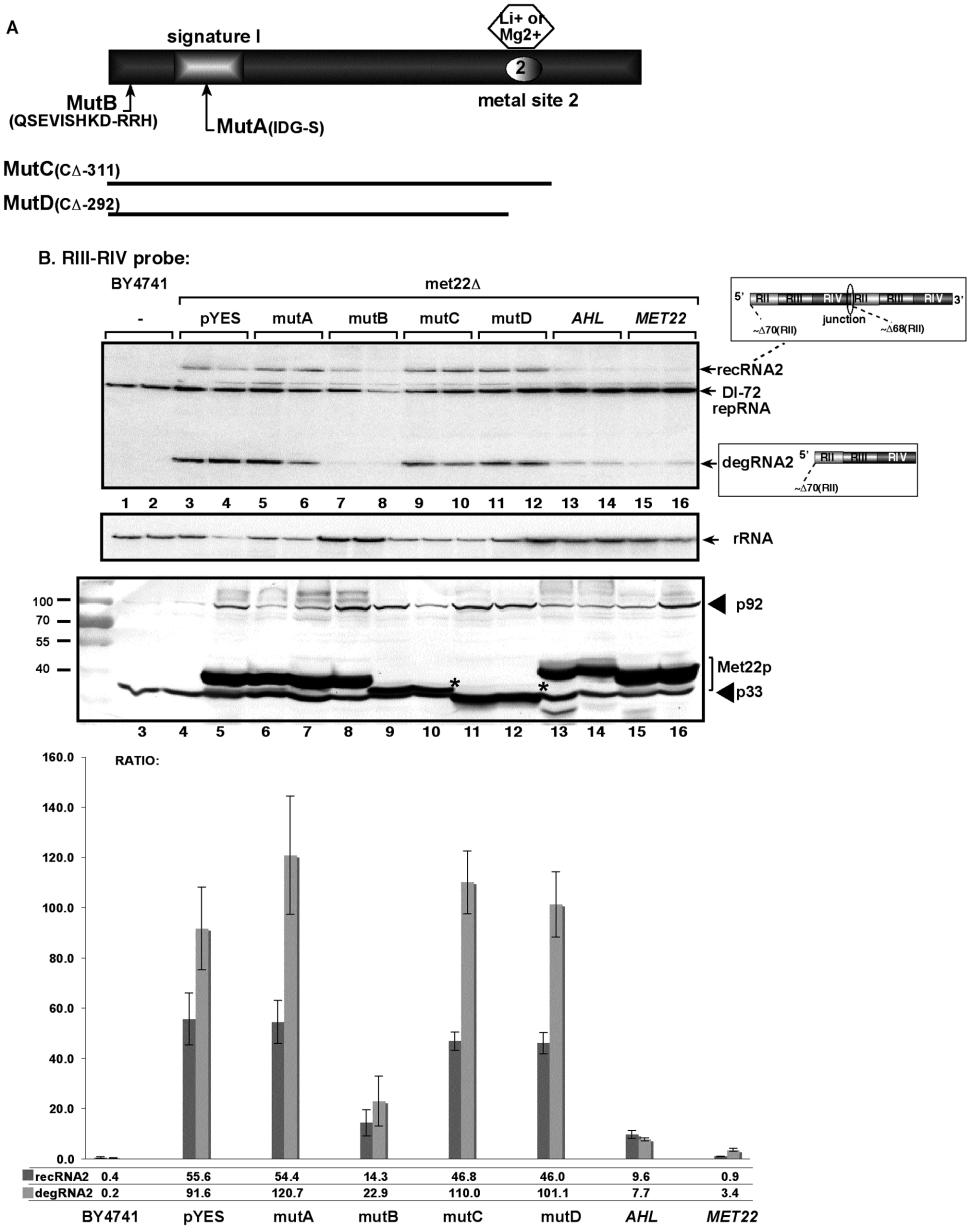
The nucleotidase function of Met22p is important for TBSV RNA recombination in yeast. (A) Schematic representation of Met22p and the mutants expressed in yeast. Note that the signature motif and the metal binding site are critical for Met22p function as a bisphosphate-3′-nucleotidase. (B) Top panel: Northern blot analysis testing the effect of mutations in *MET22* on the formation of TBSV recRNAs. Mutations are shown in panel A. The wt Met22p (lanes 15–16) and the various mutants were expressed from *GAL1* promoter in *met22Δ* yeast. *AHL* is the *Arabidopsis* nucleotidase similar to Met22p. Middle panel: Northern blot showing rRNA level as a loading control. Bottom panel: Western blotting to detect the 6xHis-tagged p33 and p92 replication proteins and the expressed Met22p mutants in *met22Δ* yeast. Asterisks mark the positions where p33 and the Met22p mutants migrated to similar positions in the gel. The graph at the bottom shows the ratio of recRNA and degRNA in comparison with the repRNA based on quantitative analysis of the Northern blots.

To test if the bisphosphate-3′-nucleotidase activity of Met22p is important for TBSV recRNA accumulation, we complemented *met22Δ* yeast with various Met22p mutants expressed from plasmids as shown in Fig. 2A. We found that expression of Met22p with mutations in the critical signature motif (MutA, Fig. 2A) or C-term truncated version of Met22p removing the metal-binding site required for binding to the essential Mg^2+^ ion (MutD) [38] resulted in lack of complementation, thus high TBSV recRNA level, in *met22Δ* yeast when compared with the expression of the wt Met22p (lanes 5–6 and 11–12 versus 15–16, Fig. 2B). In contrast, Met22p with mutations within a nonessential N-terminal segment (MutB, Fig. 2A) was able to efficiently suppress TBSV recRNA accumulation in *met22Δ* yeast (lanes 7–8, Fig. 2B). Altogether, these complementation data suggest that the bisphosphate-3′-nucleotidase function of Met22p is important for the RNA recombination suppressor activity of Met22p in yeast.

### Met22p affects the stability of TBSV RNA in yeast cell via its effect on the Xrn1p 5′-3′ exoribonuclease

The major function of Met22p bisphosphate-3′-nucleotidase in yeast cells is the removal of pAp and pApS products of the sulfate assimilation pathway, which are known inhibitors of Xrn1p 5′-3′ exoribonuclease [37], [39]. Interestingly, Xrn1p has been shown to decrease the stability of TBSV RNAs and suppress TBSV RNA recombination [30], [32], [33], [34]. Therefore, it is possible that deletion of *MET22* might promote TBSV RNA recombination by leading to an ∼80-fold increase in accumulation of pAp [41] and subsequent pAp-mediated inhibition of the ribonuclease activity of Xrn1p in yeast cells [37].

**Figure 3:**
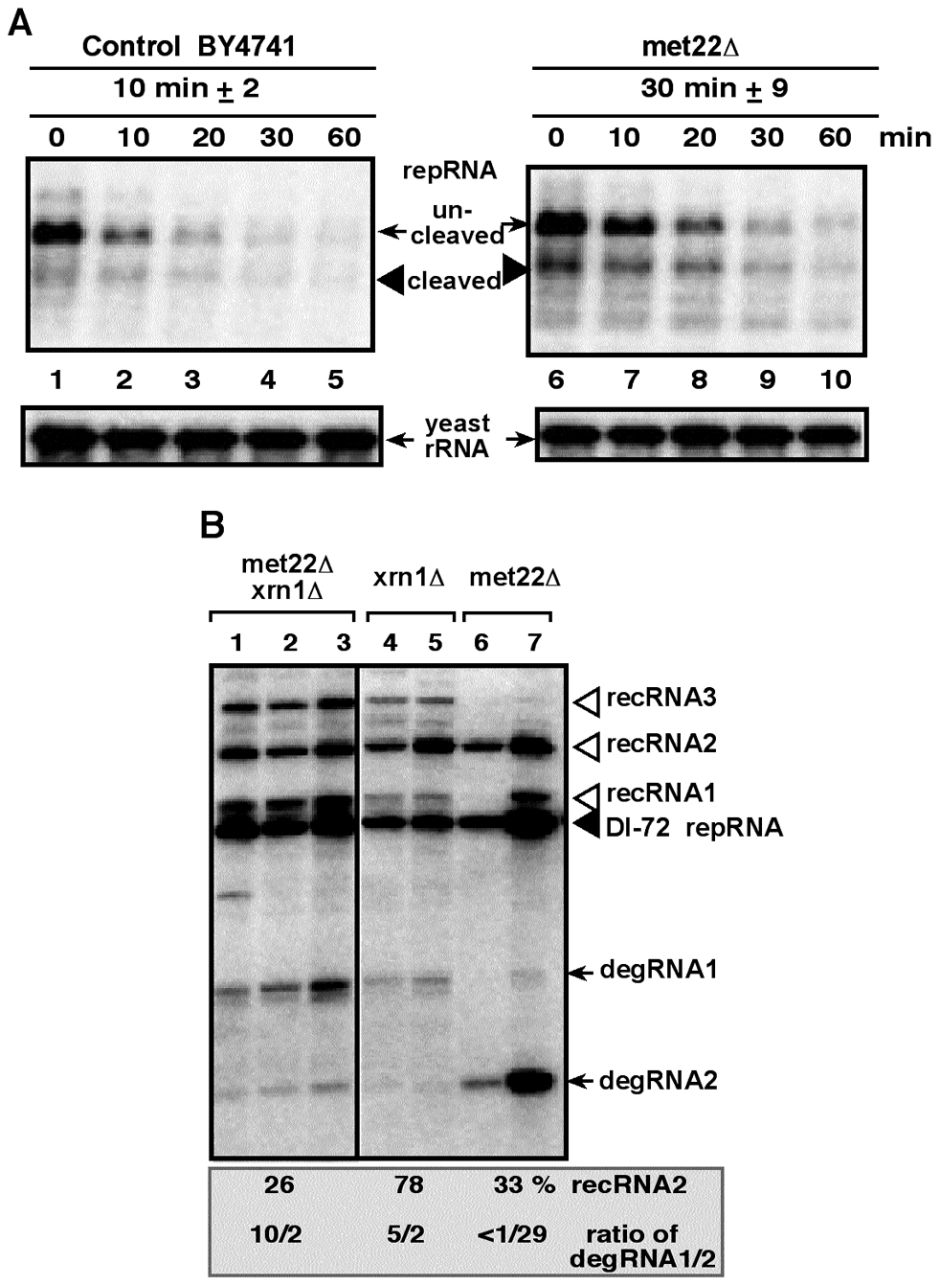
Increased stability of the TBSV repRNA in *met22Δ* yeast. (A) The DI-72 repRNA was expressed in both BY4741 parental strain and *met22Δ* strain from the *GAL1* promoter (in the absence of p92), followed by suppression of repRNA synthesis by glucose. Total RNA samples were analyzed at various time points (as shown) by Northern blotting using a DI-72(+) repRNA-specific probe. The ribozyme cleaved repRNA was quantified and the half-life of the repRNA is shown in minutes. (B) Similar profile of recRNAs and degRNAs in double deletion and in *xrn1Δ* yeasts. Northern blot analysis was performed on total RNA samples from the shown yeast strains replicating DI-72 repRNA. See further details in Fig. 1.

To test this model, we estimated the half-life of TBSV RNAs in *met22Δ* yeast. Indeed, the stability of TBSV repRNA increased by ∼3-fold in *met22Δ* when compared with the wt yeast (Fig. 3A, lanes 6–10 versus 1–5). The increased half-life for TBSV repRNA is in agreement with the possible inhibition of Xrn1p activity. In addition, the double-deletion (*met22Δ xrn1Δ*) yeast supported increased level of recRNA accumulation (by 26-fold, Fig. 3B, lanes 1–3) when compared with BY4741 (see Fig. 1, lane 6), similar to the high recombination rate in single-deletion *met22Δ* yeast or in *xrn1Δ* yeast (Fig. 3B). The profile of TBSV degRNAs accumulating in these yeasts suggest that the double-deletion strain is more similar to *xrn1Δ* than to *met22Δ* yeasts since *met22Δ xrn1Δ* yeast strain accumulates mostly the longer degRNA1 product (Fig. 3B). It is proposed that the degRNA1 product is due to a cleavage by an endoribonuclease [34]. On the contrary, *met22Δ* yeast accumulates mostly the shorter degRNA2 product, suggesting that a limited 5′-to-3′ degradation of degRNA1 by the incompletely inhibited Xrn1p nuclease still takes place in *met22Δ* yeast to give rise to degRNA2. Also, over-expression of Met22p in *xrn1Δ* yeast did not result in decreased level of recRNA accumulation (not shown), unlike when Met22p was expressed in the *met22Δ* yeast strain (Fig. 2B, lanes 15–16). Altogether, these data support the model that *MET22* and *XRN1* are both inhibitors of TBSV recombination and they act in the same pathway.

### Salt-stress caused by LiCl and NaCl treatments affect the accumulation of TBSV recRNAs in yeast cells

**Figure 4:**
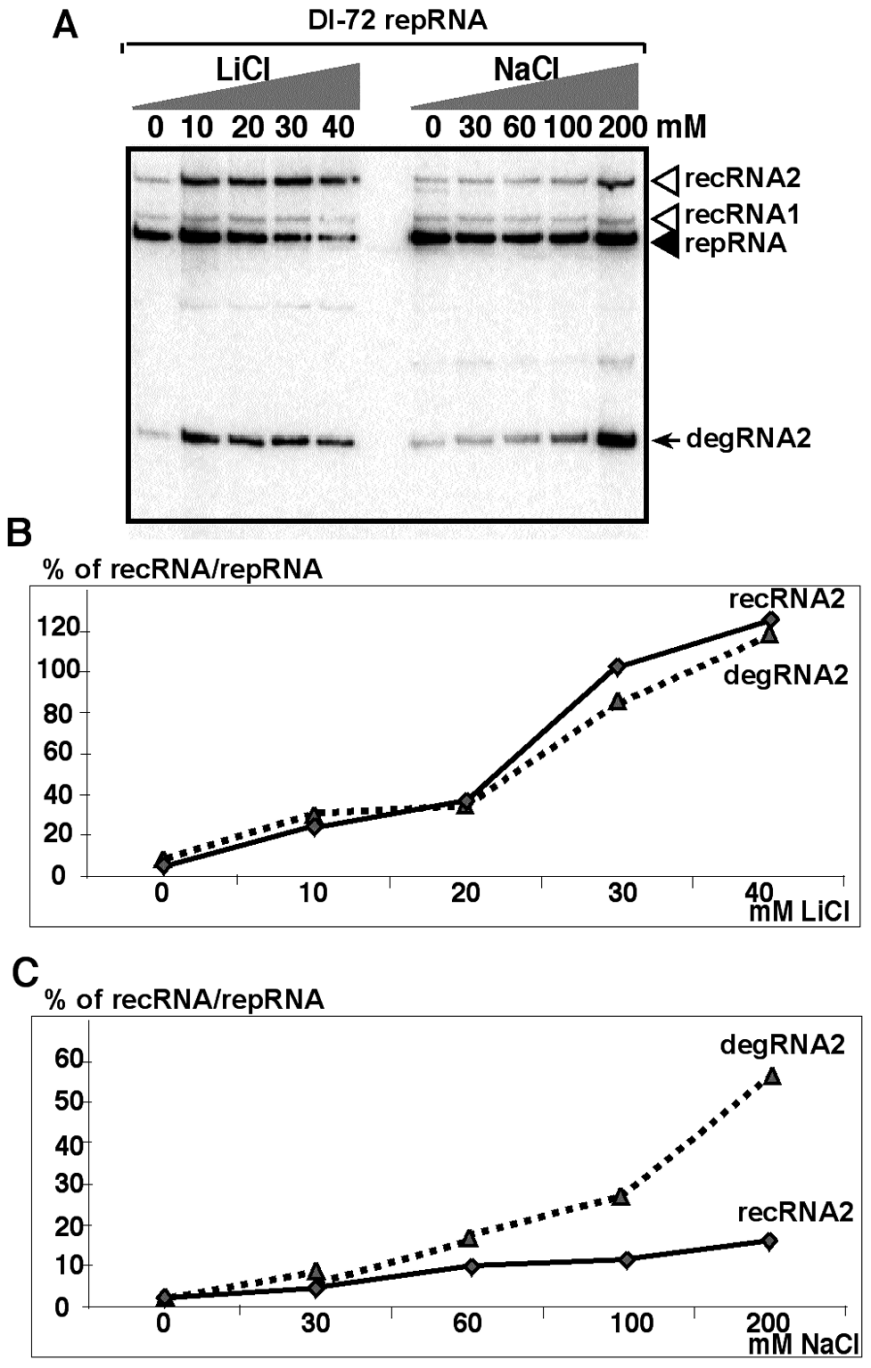
Salt-stress caused by LiCl and NaCl treatments enhances the formation and accumulation of recRNAs and degRNAs in yeast. (A) Northern blot analysis of total RNA samples from yeast replicating TBSV DI-72 repRNA. Yeast cultures were treated with the shown concentration of LiCl or NaCl. Samples were taken 24 hours after launching TBSV repRNA replication. Note that the repRNA was expressed from the *GAL1* promoter only for the first 6 hours. (B) The percent of recRNA accumulation in comparison with the repRNA (100% in each sample) in BY4741 yeast strain expressing DI-72 repRNA after treatment with LiCl. (C) The percent of recRNA accumulation in comparison with the repRNA (100% in each sample) in yeast (*gcn4Δ*) expressing DI-72 repRNA after treatment with NaCl.

Since it has been documented that salt-stress inhibits the activity of Met22p [37], [45], we tested the accumulation of recRNAs in BY4741 yeast treated first with various amounts of LiCl. We found that 20 and 40mM LiCl increased TBSV recRNA levels by ∼20 and ∼80-fold for DI-72 repRNA (Fig. 4A–B) and by up to 120-fold for the recombinogenic DI-AU-FP repRNA (Fig. S1). Since the accumulation of degRNA2 also increased remarkably in the LiCl-treated yeast (Fig. 4A–B), it is likely that the observed effect of LiCl is due to its inhibition of the Met22p-Xrn1p pathway. To obtain evidence that the above LiCl treatment indeed affects the activity of cellular 5′-3′ exoribonucleases, such as Xrn1p (cytosolic) and Rat1p (nucleus), we tested the accumulation of the nondegraded ITS1 region of pre-ribosomal RNA (Fig. S2) [37]. As expected, LiCl treatment increased the accumulation of pre-ribosomal RNA carrying the ITS1 region by ∼7-fold (Fig. S2), which is indicative of reduced level of Xrn1p and Rat1p nuclease activities in yeast cells.

Second, we tested the effect of NaCl treatment of yeast cells and found ∼7-fold increase for TBSV recRNA levels (Fig. 4A, C). The accumulation of degRNA2 also increased by ∼25-fold in the NaCl-treated yeast (Fig. 4A,C), suggesting that degradation of TBSV RNAs is decreased by NaCl due to inhibition of the Met22p-Xrn1p pathway.

### Inhibition of Met22p and Xrn1p by LiCl and pAp affects the accumulation of TBSV repRNAs and recRNAs in a cell-free extract

**Figure 5:**
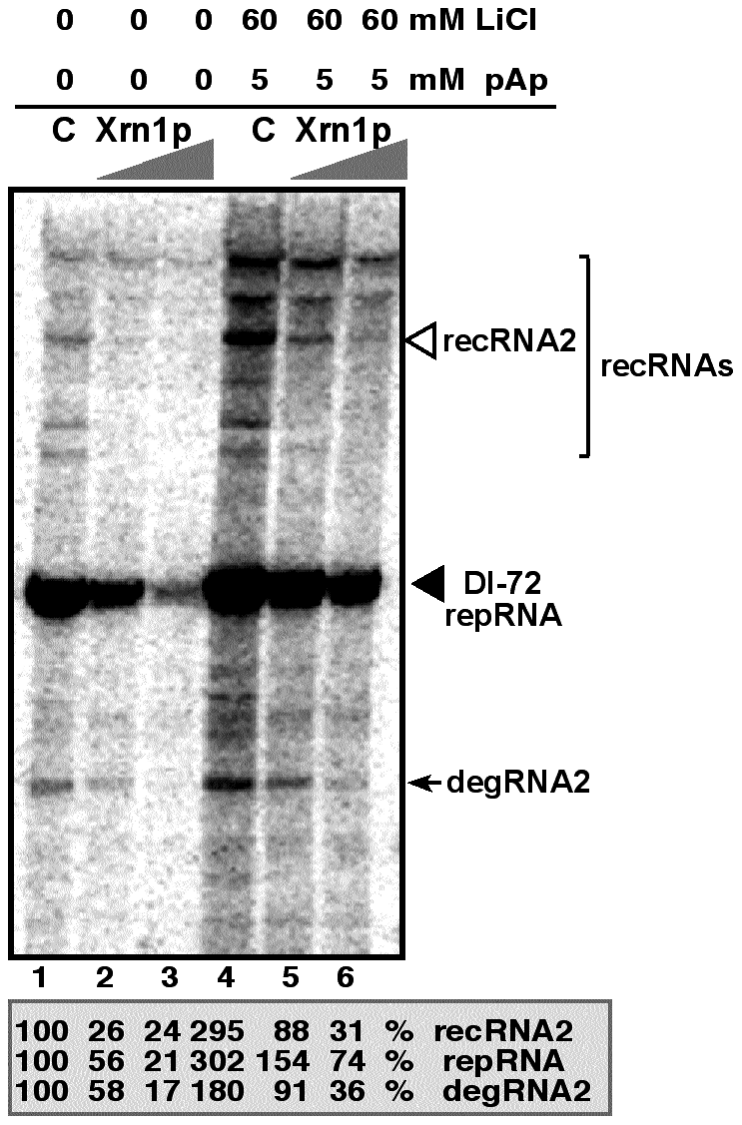
LiCl and pAp inhibit the activity of Xrn1p leading to increased accumulation of TBSV recRNA and degRNA in the cell-free yeast extract supporting TBSV RNA replication. PAGE analysis of the ^32^P-labeled RNA products obtained when the 621 nt DI-72(+) repRNA was added to the cell-free extract in the absence (lanes 1 and 4) or in the presence of TAP-affinity purified recombinant Xrn1p (1 µl and 2 µl of 0.05 mg/ml, lanes 2/5 and 3/6, respectively). The cell-free extract was obtained from yeast expressing p33 and p92^pol^ replication proteins. The slow migrating recRNAs are bracketed, whereas the partially degraded degRNA product is pointed at by an arrow. LiCl and pAp were added to the cell-free assay as shown. The percentage of repRNA, recRNA (the most abundant species, third band from the top) and degRNA2 was measured in comparison with the corresponding RNAs in the control sample (lane 1). Note that the cell-free extract contains endogenous Met22p and Xrn1p whose activities are inhibited by LiCl and pAp, leading to increased accumulation of various TBSV RNAs in lane 4.

One of the advantages of studying viral RNA recombination and replication with TBSV is the availability of a yeast-based cell-free (CFE) assay capable of supporting the in vitro assembly of the viral replicase complex, including one full replication cycle of the TBSV repRNA [31], [46], [47]. Inhibition of the endogenous Met22p and Xrn1p present in the CFE obtained from wt BY4741 yeast by 60 mM LiCl and 5 mM pAp led to ∼3-fold increase of both repRNA and recRNA accumulation (Fig. 5, lanes 1 versus 4). The amount of degRNA also increased by ∼2-fold. However, adding purified recombinant Xrn1p to the above CFE containing the inhibitors, led to ∼10-fold inhibition of recRNA accumulation (Fig. 5, lanes 4 versus 6), while the accumulation of repRNA and degRNA decreased by ∼4 and ∼5-fold, respectively. When compared with the control sample, 60 mM LiCl and 5 mM pAp inhibitors did decrease the suppressor activity of the exogenous Xrn1p by ∼3-fold in TBSV recombination and replication (compare lanes 5 with 2, Fig. 5). Altogether, the in vitro data strongly support the role of LiCl and pAp in TBSV RNA recombination and replication by inhibiting the recombination suppressor activity of Xrn1p.

### Treatment of plant protoplasts with LiCl and pAp increases the accumulation of TBSV recRNAs

**Figure 6:**
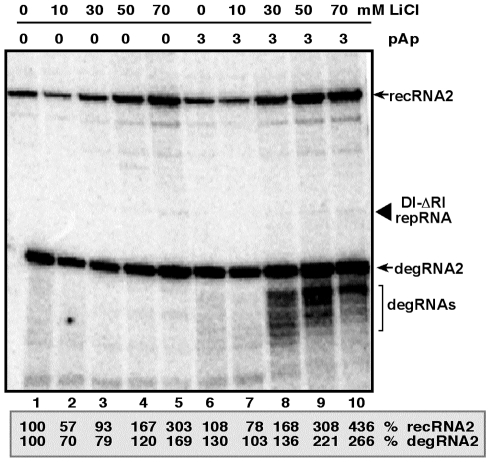
Increased accumulation of TBSV recRNA in *N. benthamiana* protoplasts by treatment with LiCl and pAp. Northern blot analysis was used to detect the accumulation levels of TBSV repRNA, recRNAs and degRNAs based on a 3′-specific RIII/IV probe. *N. benthamiana* protoplasts were treated with the shown concentrations of LiCl and pAp immediately after electroporation of the repRNA and the helper CNV genomic RNA. The samples were harvested 20 hours after electroporation. Note that the highly recombinogenic TBSV-ΔRI repRNA contains only three regions (RII-to RIV) derived from DI-72 repRNA. The recRNAs are derived via template-switching recombination occurring between two 5′ truncated DI-ΔRI repRNA. Relative accumulation levels of the most abundant recRNA2 and degRNA2 are shown (100% was chosen for the samples not treated with LiCl and pAp). Note that TBSV-ΔRI repRNA accumulates to low level.

To test if plants have a pathway similar to Met22/Xrn1 pathway in yeast that can affect recombination of TBSV, first we used LiCl and pAp inhibitors in *Nicotiana benthamiana* protoplasts electroporated with DI-ΔRI repRNA that lacks the 5′ terminal 169 nt from the wt DI-72 repRNA and can recombine efficiently in plant protoplasts and yeast [31], [32], [33]. We found that LiCl treatment increased TBSV recRNA and degRNA accumulation by ∼3- and ∼1.5-fold, respectively, after 24 hours of incubation (Fig. 6, lanes 5 versus 1), while pAp treatment alone had no significant effect on TBSV recRNA and degRNA accumulation (lane 6). However, the largest stimulatory effect on TBSV recRNA and degRNA accumulation was obtained by the combined use of LiCl and pAp, leading to ∼4.5- and 2.5-fold increase, respectively (lane 10). Overall, the data from protoplasts suggest that plant cells also have a Met22/Xrn1-like pathway that is inhibited by LiCl and pAp, thus resulting in increased level of TBSV recombination.

### Silencing of *MET22* homologs in plants increases the accumulation of TBSV repRNAs and recRNAs

To examine if a plant nucleotidase analog of the yeast *MET22* gene can also affect TBSV RNA recombination, first, we expressed the *Arabidopsis AHL* nucleotidase/phosphatase gene [48] in *met22Δ* yeast. Interestingly, *AtAHL* reduced the accumulation of TBSV recRNA and degRNA by 5- and 10-fold, respectively (lanes 13–14, Fig. 2B), confirming that a plant analog of the yeast *MET22* gene can also suppress TBSV recombination.

**Figure 7:**
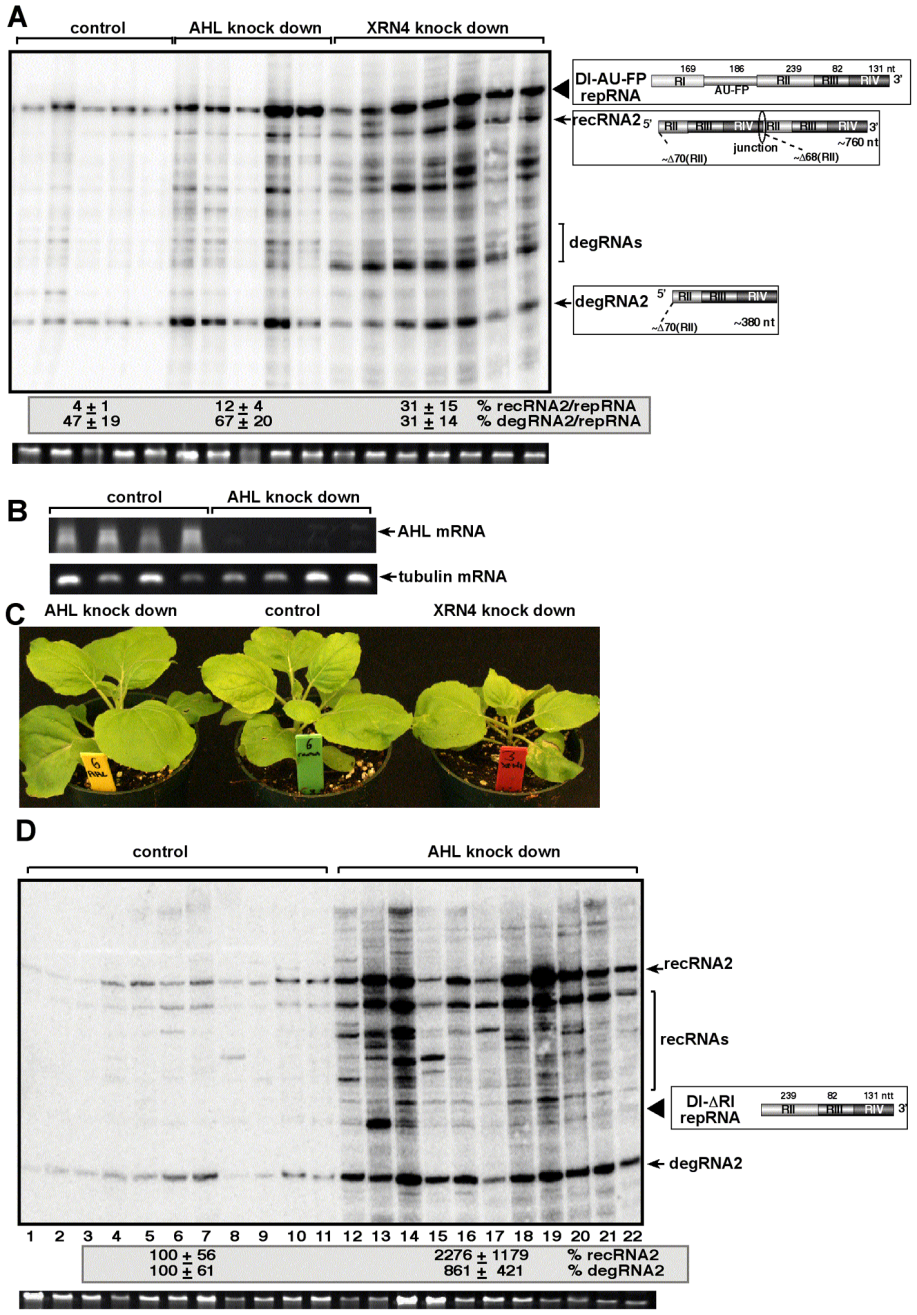
The role of *AHL1* phosphatase in TBSV RNA recombination. (A) Increased accumulation of TBSV recRNAs and degRNAs in *AHL* and *XRN4* knockdown *N. benthamiana* plants 3 days post-inoculation, based on Northern blot analysis. VIGS was performed via agroinfiltration of *Tobacco rattle virus* (TRV) vectors carrying either *AHL1* or *XRN4* sequences or the TRV empty vector (as a control). Co-agroinfiltration to express TBSV DI-AU-FP repRNA together with CNV gRNA was done 9 days after silencing of *AHL* and *XRN4* expression by agroinfiltration. Ribosomal RNA is shown as a loading control at the bottom of the panel. Note that the *AHL1* gene is the ortholog of yeast *MET22*. (B) Semi-quantitative RT-PCR analysis of the accumulation of *AHL1* mRNA in *AHL1* knockdown *N. benthamiana* plants and in the control plants, which were agroinfiltrated with the TRV vector, 9 days after agroinfiltration. RT-PCR analysis of the tubulin mRNA from the same samples serves as a control. (C) The phenotype of the *AHL* and *XRN4* knockdown *N. benthamiana* plants 9 days after agroinfiltration. (D) Accumulation of the highly recombinogenic TBSV DI-ΔRI repRNA and newly formed recRNAs in *AHL1* knockdown *N. benthamiana* plants 3 days post-inoculation, based on Northern blot analysis. The original DI-ΔRI repRNA and the most frequently generated recRNA are shown schematically on the right. See further details in Panel A.

To test if silencing of the *AHL* gene in *N. benthamiana* could influence TBSV recombination, we agroinfiltrated *N. benthamiana* leaves with plasmids expressing *Cucumber necrosis virus* (CNV), which can be used as a helper tombusvirus, and the highly recombinogenic TBSV DI-AU-FP RNA after knocking down the level of *NbAHL* mRNA via gene silencing (Fig. 7B). The accumulation of TBSV recRNAs was increased by ∼3-fold in the agroinfiltrated leaves of the *NbAHL* knockdown plants (Fig. 7A), which is less than ∼8-fold increase observed in *XRN4* (the homolog of the yeast *XRN1*) [32] knockdown plants. Knocking down the expression of *NbAHL* did not affect the growth of *N. benthamiana*, while the *XRN4* knockdown plants showed some stunting (Fig. 7C).

To further test TBSV recombination in *AHL* knockdown plants, we used agroinfiltration with plasmids expressing DI-ΔRI repRNA in combination with the CNV helper virus to launch replication in the silenced leaves. Subsequent analysis of TBSV RNA levels revealed that the levels of recRNAs and degRNAs were increased by ∼22- and ∼9-fold, respectively (Fig. 7D). Thus, two different TBSV repRNAs showed high frequency recombination in plants silenced for *AHL* nucleotidase/phosphatase, confirming that plant *AHL* plays a comparable role in TBSV recombination to the yeast *MET22* nucleotidase.

**Figure 8:**
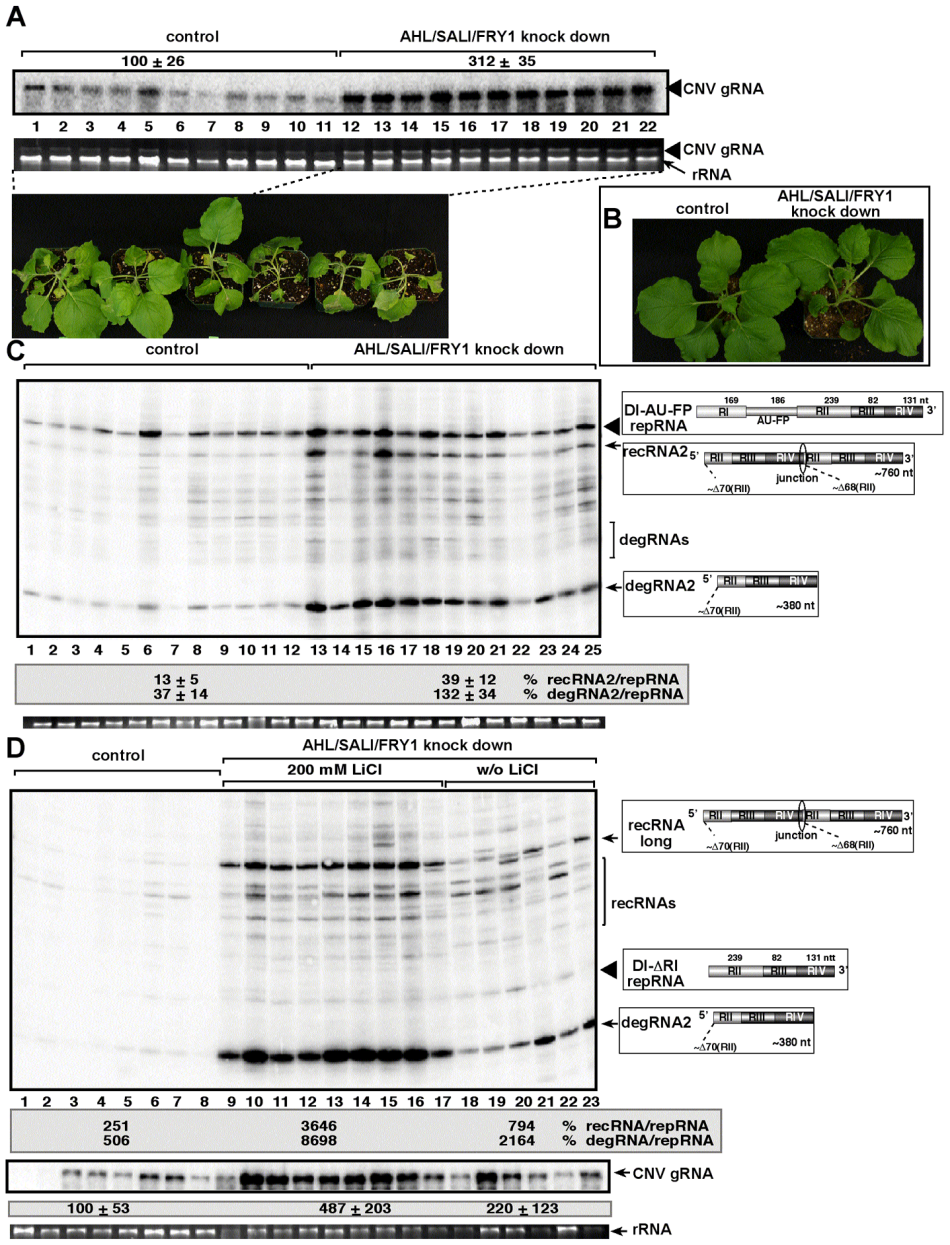
Increased accumulation of tombusvirus RNAs in of *AHL1/SAL1/FRY1* triple-gene knock down *N. benthamiana* plants. (A) Increased accumulation of tombusvirus (*Cucumber necrosis virus*, CNV, closely related to TBSV) genomic RNA in *AHL1/SAL1/FRY1* knockdown *N. benthamiana* plants 3 days post-inoculation, based on Northern blot analysis. The bottom image shows the enhanced symptoms on *AHL1/SAL1/FRY1* knockdown *N. benthamiana* plants 5 days post-inoculation with CNV. (B) The phenotype of the *AHL1/SAL1/FRY1* triple-gene knockdown *N. benthamiana* plants 9 days after agroinfiltration with the VIGS vectors. (C) Increased accumulation of TBSV recRNAs and degRNAs derived from TBSV DI-AU-FP repRNA in *AHL1/SAL1/FRY1* knockdown *N. benthamiana* plants 3 days post-inoculation, based on Northern blot analysis. See further details in Fig. 8A. (D) LiCl treatment enhances the accumulation of TBSV recRNAs and degRNAs derived from TBSV DI-ΔRI repRNA in *AHL1/SAL1/FRY1* knockdown *N. benthamiana* plants 3 days post-inoculation, based on Northern blot analysis. The panel in the middle shows Northern blot analysis of the accumulation of CNV genomic RNA, the helper virus in the above plants. See further details in Fig. 8A.

Since there are at least three Met22-like nucleotidases in *Arabidopsis*, such as *AHL*, *SAL1* and *FRY1* [49], [50], we decided to knockdown the expression levels of all three genes simultaneously. The accumulation of the CNV helper virus increased by ∼3-fold in the triple-nucleotidase gene knockdown plants, which died ∼2–3 days faster than the control plants after co-agroinfiltration with plasmids expressing both CNV helper virus and the TBSV DI-AU-FP repRNA (Fig. 8A). The uninoculated triple knockdown plants showed slight stunting, but the individual leaves were actually larger than the leaves of the control plants treated with the “empty\ silencing vector (Fig. 8B).

The accumulation of TBSV recRNAs and degRNAs increased ∼3- and 4-fold, respectively, in the DI-AU-FP repRNA inoculated leaves of triple gene knockdown plants (Fig. 8C), suggesting that recombination was comparable in the *AHL*-knock down and the triple-nucleotidase gene knockdown plants.

It is likely that there is still some residual nucleotidase activity in the triple gene knockdown plants, therefore, we infiltrated 200 mM LiCl to leaves to further inhibit the nucleotidase activity in the triple-nucleotidase gene knockdown *N. benthamiana* plants. Interestingly, we observed ∼8-fold increase in TBSV recRNA and ∼4-fold increase in degRNA accumulation in the triple gene knockdown plants infiltrated with LiCl when compared to triple gene knock down plants infiltrated with water control (Fig. 8D, compare lanes 9–17 with 18–23). When compared to the control nonsilenced and untreated plants replicating CNV helper and the TBSV DI-ΔRI repRNA, the accumulation of TBSV recRNAs and degRNAs increased by ∼15- and 17-fold, respectively, in the triple gene knockdown plants infiltrated with LiCl (Fig. 8D, compare lanes 9–17 with 1–8). The accumulation of the CNV helper virus also increased by ∼5-fold in the triple gene knockdown plants infiltrated with LiCl (Fig. 8D). Overall, these data strongly support the role of plant *AHL*, *SAL1* and *FRY1* nucleotidases/phosphatases in tombusvirus recombination, replication and viral RNA degradation.

## Discussion

Viral RNA recombination plays a major role in virus evolution [3], [5], [6]. In spite of the possible significance, we know little about the roles of host and environmental factors in viral RNA recombination [51]. In this work, using TBSV and yeast as a model host, we demonstrate that Met22/Xrn1 pathway and environmental factors affecting this pathway, namely salt-stress caused by LiCl and NaCl, plays a role in viral RNA recombination. In vitro experiments with a cell-free extract from yeast revealed that the combined use of LiCl, an inhibitor of Met22p bisphosphate-3′-nucleotidase, and pAp, an inhibitor of Xrn1p 5′-3′ exoribonuclease, could promote TBSV RNA recombination (Fig. 5). Since there is only a single cycle of RNA replication in the CFE, the fact that RNA recombinants accumulate at a detectable level in vitro suggests that LiCl and pAp are potent inducers of viral RNA recombination. These compounds also reduce the complete degradation of the viral RNA and increase the accumulation of the original repRNA by inhibiting the activity of the Xrn1p ribonuclease in the CFE. Xrn1p ribonuclease is a major enzyme controlling degradation of the tombusvirus RNA, which is uncapped at the 5′ end [52]. Thus, inhibition of the activity of the 5′-3′ exoribonuclease leads to increased levels of partially degraded TBSV RNA products, which then could affect (i) the frequency of RNA recombination by serving as intermediate templates during recombination events [30], [31], [34], (ii) facilitate the formation of defective interfering RNAs [52], and (iii) possibly alter the fitness of viral populations.

Genetic experiments in yeast model host also supported that *MET22* affects viral RNA recombination via *XRN1*. For example, deletion of *MET22* increased the half-life/stability of the TBSV RNA by three fold, suggesting that the activity of the Xrn1p ribonuclease, the major factor involved in TBSV RNA degradation in yeast [32], [33], [34], is inhibited via the pAp substrate of Met22p [37], [39]. Moreover, the double deletion (*met22Δ xrn1Δ*) strain behaved as the single deletion (*xrn1Δ*) strain in the TBSV recombination assay (Fig. 3). Also, the profile of the partially degraded viral RNA products in the single and double deletion strains was similar (Fig. 3). Therefore, we propose that Met22p is a suppressor of TBSV recombination via its regulatory function of Xrn1p activity.

We also provide evidence that comparable pathway to the Met22/Xrn1 pathway of yeast also regulates TBSV RNA recombination in plant cells. Addition of LiCl to the *N. benthamiana* protoplast media or the combined use of LiCl and pAp both resulted in increased TBSV RNA recombination and led to higher levels of partially degraded TBSV RNAs (Fig. 6). Also, expression of the Ahl nucleotidase/phosphatase from *Arabidopsis*, a yeast *MET22* analog, suppressed TBSV RNA recombination and decreased the accumulation of the partially degraded viral RNAs in *met22Δ* strain. Silencing of the expression of *AHL* gene alone, or triple-gene silencing of *AHL*, *SAL1* and *FRY1* nucleotidase/phosphatases in *N. benthamiana* increased the level of TBSV RNA recombinants in plant leaves. The most pronounced increase in accumulation of TBSV RNA recombinants was seen in the triple-gene silenced plants treated with LiCl, suggesting that the combined effect of genetic and environmental factors could be critical in regulation of the rate of viral RNA recombination.

**Figure 9:**
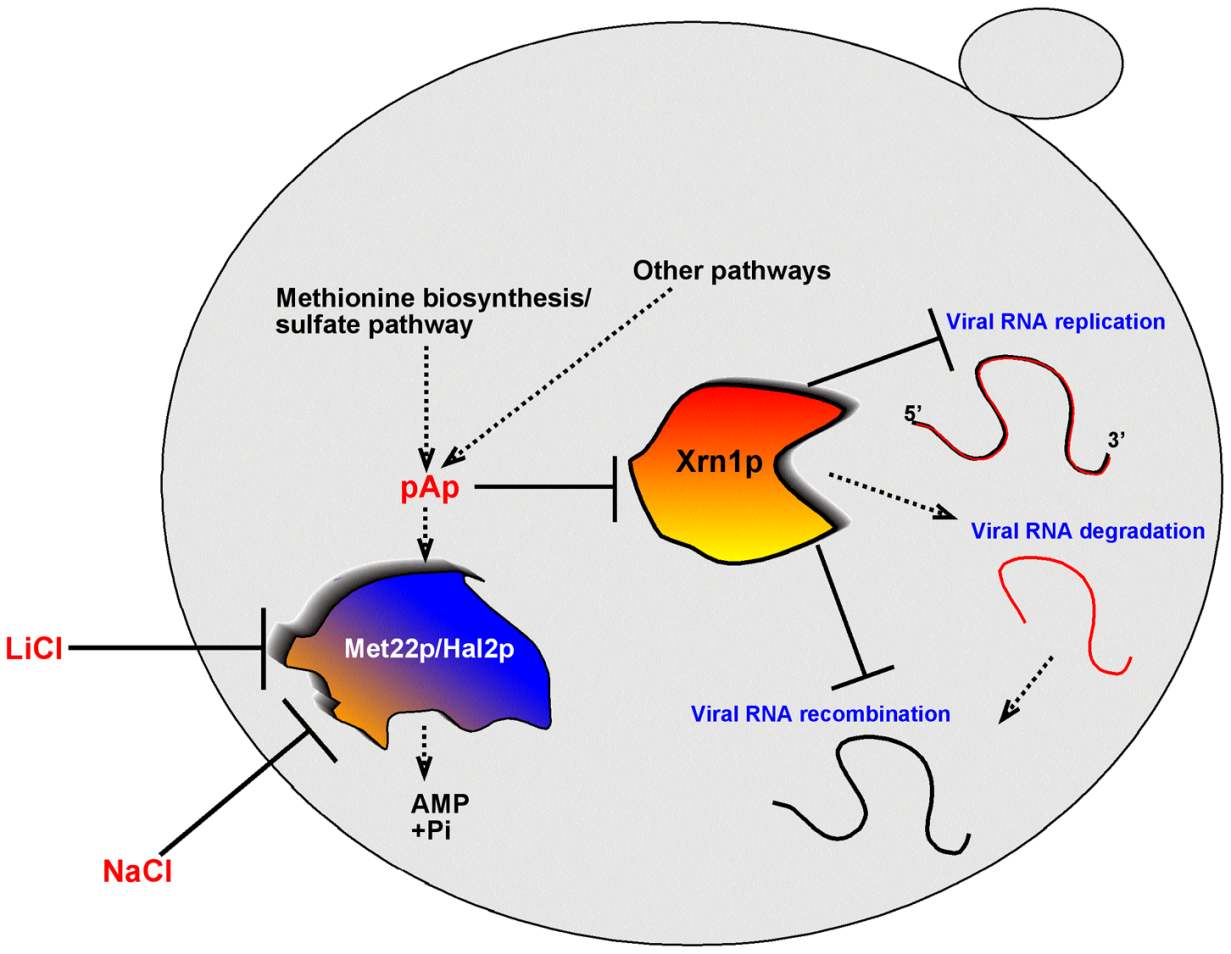
A model explaining the role of the yeast Met22p bisphosphate-3′-nucleotidase in TBSV recombination, based on regulation of the activity of Xrn1p 5′-to-3′ exoribonuclease in the cytosol. In the presence of Met22p, the amounts of cytosolic pAp and other pNp are low, due to the nucleotidase activity of Met22p. Therefore, the activity of Xrn1p is high, which then leads to rapid degradation of TBSV degRNAs (intermediates of RNA recombination) and a low frequency of RNA recombination. In the absence of Met22p or when the activity of Met22p is inhibited by external LiCl or NaCl, the pAp/pNp concentration increases in the cytosol. The high pAp/pNp levels result in low activity of Xrn1p, which in turn, leads to increased accumulation of TBSV degRNAs and a high recombination frequency. In addition, the overall level of TBSV repRNA also increases due to the increased half-life of the viral RNA when the activity of Xrn1p is inhibited by elevated pAp/pNp levels.

Based on the known biochemical functions of Met22p, as well as the presented in vitro and in vivo results on TBSV recombination, we propose that Met22p (*AHL*, *SAL1* and *FRY1* nucleotidases in *N. benthamiana*) regulates TBSV RNA recombination and degradation of TBSV repRNA via affecting pAp level in cells (Fig. 9). Moreover, Met22p also affects the level of other biphosphorylated nucleotides (pNps, such as pCp, pGp, pTp, pUp and pIp) generated by various pathways in cell [40], raising the possibility that other pNps might also affect viral RNA recombination. In the presence of active Met22p in cells, pAp/pNp level is low, thus allowing high activity of Xrn1p ribonuclease, which in turn, removes the partially degraded TBSV RNA products from cells [32], [34]. The partially degraded TBSV RNA products are generated by an unidentified endoribonuclease(s) and rapidly degraded by Xrn1p exoribonuclease in the parental BY4741 yeast [34]. However, as shown earlier, the accumulation of the partially degraded TBSV RNA products in *xrn1Δ* yeast leads to high frequency recombination due to the use of these degRNAs in the recombination events by the viral replicase [32], [34]. We show that deletion of *MET22* likely prevents the rapid and complete degradation of repRNAs due to the accumulation of pAp substrate of Met22p, which inhibits the activity of Xrn1p 5′-3′ exoribonuclease [37], [39]. Therefore, the high level of TBSV degRNAs in *met22Δ* cells will promote high frequency RNA recombination as well as decrease the rate of their degradation. In addition, environmental factors, such as LiCl and NaCl causing salt-stress, could affect TBSV RNA recombination by inhibiting the activity of Met22p in yeast cells (Fig. 9). Overall, our results suggest that environmental and host factors could play an important role in viral RNA recombination and evolution.

## Materials and Methods

### Yeast strains and expression plasmids

*S. cerevisiae* strains BY4741 (MATa his3Δ1 leu2Δ0 met15Δ0 ura3Δ0), *met22Δ*, *gcn4*Δ, and *xrn1*Δ were obtained from Open Biosystems. To express wt and mutated Met22p proteins in *met22Δ* yeast, we made pYES-Met22 plasmid expressing a 6xHis-Met22p (tag is present at the N- terminus) under the control of a *GAL1* promoter. The cDNA of *MET22* was amplified with primers #2177 and #2178 (Table S1) and cloned at the *KpnI/XhoI* sites of pYES2/NT. To obtain pYES-MetA and pYES-MetB, expressing mutants MetA and MetB (Fig. 2A), the *MET22* sequence was amplified with primers #2584/#2178 and #2585/#2178, respectively, and the PCR products were cloned to either *BamHI/XhoI* linarized pYES-Met22 or *EcoRI/XhoI* linarized pYES-Met22. pYES-MetC and pYES-MetD, expressing mutants MetC and MetD, were obtained by PCR with primers #2586/#2177 and #2587/#2177, respectively, and the PCR products were digested with *KpnI/XhoI* and cloned into *KpnI/XhoI* linarized pYES plasmid. For generating the expression vector pYES-AHL*At*, the cDNA sequence of *AHL* was generated from *Arabidopsis* total RNA extract using RT-PCR with primers #2588/#2589 and then the RT-PCR product was cloned to the *BamHI/XhoI* site of pYES2/NT.

Yeast strain *met22Δxrn1Δ* was obtained by homologous recombination of yeast strain *xrn1Δ* (Open Biosystems) with the PCR product of primers #2581/#2590 and pFA6-hphNT1 [44] as a template for hphNT1 selection. Correct deletion of the *MET22* gene was verified by PCR with primers #2501/#2591.

Yeast strain *Gals-met22* was obtained based on homologous recombination. Briefly, the BY4741 yeast strain was transformed with the PCR product made by using primers #2581/#2583 and pYM-N30 [44] as a template for kanMX4 selection. Correct integration of the *GALS* sequence in strain *Gals-met22* strain was verified with primers KanB (Open biosystems)/#2591.

### Yeast transformation and cultivation

Yeast strains were co-transformed with indicated plasmids or PCR products by using the lithium acetate/ssDNA/polyethylene glycol method [53], and transformants were selected by complementation of auxotrophic markers.

The yeast strain Gals-met22 was co-transformed with pGBK-His33/DI72/CUP1 and pGAD-His92/CUP1 [27], [31]. The transformed yeast strains were grown at 29°C in SC-UHL (synthetic complete media without uracil, histidine and leucine) with 2% glucose containing 50 µM CuSO_4_ until reaching cell density of 0.6 OD_600_. Then yeast cultures were resuspended in SC-UHL with galactose medium containing 50 µM CuSO_4_. Yeast was grown at 29°C for indicated timepoints before collecting for Northern analyses.

For the complementation study (Fig. 2), yeast was co-transformed with pGBK-His33/DI72/CUP1, pGAD-His92/CUP1 and the indicated Met22p mutants in pYES. The transformed yeast strains were pre-grown at 29°C in SC-UHL with 2% glucose until reaching cell density of 1.0 OD_600_. Then yeast cultures were diluted to 0.1 OD_600_ in SC-UHL with galactose medium containing 50 µM CuSO_4_ to launch TBSV repRNA replication and to co-express the Met22p mutants. Yeast was grown at 29°C for 24 hours before sample collection for analyses. The statistical analysis was performed using AVEDEV (average of absolute deviations of data points) program in Microsoft Excel (version 2008 for Mac) based on 12–24 independent samples.

For the RNA stability studies (Fig. 3A), yeast strains BY4741 and *met22Δ* were transformed with pYC2-DI72 [43]. The transformed yeast strains were grown at 29°C in SC-U (synthetic complete without uracil) with 2% galactose. After 20 h, the cultures were re-suspended in SC-U supplemented with 2% glucose and collected after indicated time-points.

For the analysis of TBSV repRNA replication and recombination in *met22Δxrn1Δ*, *xrn1Δ* and *met22Δ* yeast strains, they were co-transformed with pGBK-His33/CUP1 [28], pGAD-His92/CUP1 and pYC2-DI72. The transformed yeast strains were pre-grown at 29°C in SC-UHL supplemented with 2% glucose until reaching cell density of 1.0 OD_600_.

The pre-grown yeast cultures were diluted to 0.1 OD_600_ in SC-ULH medium supplemented with 2% galactose and 50 µM CuSO_4_ to launch TBSV repRNA replication. After 6h at 23°C, the cultures were collected and re-suspended in SC-UHL supplemented with glucose and 50 µM CuSO_4_. The yeast cultures were grown for additional 18 hours at 23°C before sample collection for analyses.

For the LiCl treatment (Fig. 4), yeast strain BY4741 was co-transformed with pGBK-His33/CUP1, pGAD-His92/CUP1 and pYC2-DI72 or pYC2-DI-AU-FP, respectively. For the NaCl treatment (Fig. 4), we used yeast strain *gcn4Δ*, because it has been shown that NaCl treatment increases indirectly the activity of *GCN4* transcription factor, which then upregulates the expression of *MET22* [45]. The transformed yeast strains were pre-grown at 29°C in SC-UHL supplemented with 2% glucose until reaching cell density of 1.0 OD_600_. The pre-grown yeast cultures were diluted to 0.1 OD_600_ in SC-UHLM+A (synthetic complete without uracil, histidine, leucine, methionine, supplemented with 5g/l ammonium sulphate) with 2% galactose and 50 µM CuSO_4_ to launch TBSV repRNA replication and the indicated amount of LiCl or NaCl (Fig. 4, and S1). After 6h culturing, yeast cells were collected and re-suspended in SC-UHLM+A supplemented with glucose, 50 µM CuSO_4_ and LiCl or NaCl. The yeast cultures were grown for additional 18 hours before sample collection for analyses.

### Plant protoplast experiments

T7 transcripts of DI-ΔRI and CNV RNAs were obtained as described previously [34]. Isolation, electroporation and culturing *N. benthamiana* protoplasts was done as described [54]. Briefly, 6 µg DI-ΔRI RNA together with 5 µg CNV genomic RNA transcripts were used for co-electroporation of 30×10^5^ *N. benthamiana* protoplasts in 1ml electroporation buffer [54]. After electroporation, protoplasts were resuspended in 5 ml SP (34.2 g/l sucrose, 0.58 g/l MES, 72.8 g/l mannitol, pH 5.8) with indicated amounts of LiCl and pAp (adenosine 3′-5′ biphosphate, Sigma). Protoplasts were incubated in the dark for 20 h at room temperature followed by RNA extraction and Northern blot analysis as described previously [54].

### Virus-induced gene silencing of nucleotidase genes in *N. benthamiana* plants

The virus-induced gene silencing (VIGS) assay was described previously [31], [55], [56]. The *N. benthamiana* sequences were obtained by a BLAST search using the *Arabidopsis thaliana AHL*, *SAL1* and *FRY1* gene sequences in *Solanaceae Genomics Resource* mRNAs database from the J. Craig Venter Institute. *NbAHL* correspondents to clone EB432053, *NbSAL1* to TA11598 and *NbSAL2* to BP135480, respectively.

The VIGS vectors, pTRV2-AHLNb, pTRV2-Sal1Nb and pTRV2-FRY1Nb, were obtained by RT-PCR with primers #2191/#9192 for *NbAHL*, #2935/#2937 for *NbSAL1* and primers #2932/#2933 for *NbFRY1*. Nine days after the VIGS treatment (using one or all three VIGS vectors together with pTRV1) [31], [56], the level of *NbAHL* mRNA was determined by RT-PCR with primers #2940/#2941. We used the level of tubulin mRNA as a control by RT-PCR using primers #2859/#2860. Subsequently, the silenced leaves were co-agroinfiltrated with pGD-CNV and pGD-ΔRI, or pGD-CNV and pGD-DI-AU-FP. One day after agroinfiltration, selected leaves were infiltrated with 200mM LiCl. Leave samples were collected of the agroinfiltrated leaves four days after agroinfiltration, followed by total RNA extraction and Northern blot analysis as described [32].

### Cell-free TBSV replication and recombination assay

TAP-tagged purified Xrn1p [34] or Xrn1p-His (from A.W. Jonson) [57] was added to yeast cell-free extract, which were programmed with 1 µg of DI-72 or DI-ΔRI repRNAs as described [47]. The in vitro replication assay was performed for 4 hours at 25°C. Total RNA was extracted and loaded on a 5% polyacrylamide gel (PAGE) containing 8 M urea. The ^32^P-labeled bands were imaged with a Typhoon 9400 Imager (GE Healthcare) [47].

### Tombusvirus RNA analysis

TBSV RNA replication and recombination was analyzed using total RNA obtained from yeast or plants. Northern blot analysis were performed as described previously [33]. Briefly, for detection of DI-72 repRNA and its derivatives, including recRNAs, we prepared a ^32^P-labeled region III+IV probe with T7 transcription using PCR amplified DNA obtained with primers #2754 and #2755 and pYC-DI72 as template. Northern blots were imaged with Typhoon (GE Healthcare) and analyzed by the ImageQuant program. Quantification was performed and the recRNA2 or degRNA2 levels were calculated in comparison to the amount of repRNA in each sample. Also, the repRNA measurements were normalized based on the ribosomal RNA levels in each sample.

To detect the CNV genomic RNA, we made a ^32^P-labeled complementary RNA probe with T7 transcription from PCR products obtained with primers #312/#22 (3′ end) or #1660/#20 (5′-3′ end). An RNA probe was obtained for detection of region I of DI-72 (using primers #20/#15 in PCR). To detect MET22 mRNA, we made a ^32^P-labeled complementary RNA probe obtained by T7 transcription from a PCR product using primers #2177/#2200.

## Supporting Information

Table S1 Yeast strains, expression plasmids and primers.
(0.12 MB PDF)

Figure S1 LiCl enhances the formation and accumulation of recRNAs and degRNAs in yeast. (A) Northern blot analysis of total RNA samples from yeast replicating TBSV DI-72 repRNA. BY4741 yeast cultures were treated with the shown concentration of LiCl. Samples were taken 24 hours after launching TBSV repRNA replication. Note that the repRNA was expressed from the *GAL1* promoter only for the first 6 hours. (B) The percent of recRNA accumulation in comparison with the repRNA (100% in each sample) in yeast expressing DI-72 or DI-AU-FP repRNAs, respectively. (C) Northern blot analysis of total RNA samples from yeast replicating TBSV DI-AU-FP repRNA. See further details in panel A. (D) Schematic representation of the recRNA2 formed in yeast replicating DI-AU-FP. The replicase-driven template-switching event between two repRNAs is shown with an arrow. Note that the recombinants have variable sequences at the very 5′ end and at the junction sites, thus most of the recRNAs are different from one another.
(0.64 MB PDF)

Figure S2 Increased level of accumulation of the nondegraded ITS1 region of pre-ribosomal RNA after LiCl treatment. The activity of cellular 5′-3′ exoribonucleases, such as Xrn1p (cytosolic) and Rat1p (nucleus), was inhibited by the shown amount of LiCl as described. As expected, LiCl treatment increased the accumulation of pre-ribosomal RNA carrying the ITS1 region by up to 7-fold, which is indicative of reduced level of Xrn1p and Rat1p nuclease activities in yeast cells.
(0.22 MB PDF)
