# Supplementary material for: The Combined Effect of Environmental and Host Factors on the Emergence of Viral RNA Recombinants
Source: PLoS Pathog. 2010 Oct 21;6(10):e1001156. doi: 10.1371/journal.ppat.1001156 (PMC2958810; doi:10.1371/journal.ppat.1001156)
Supplement: Table S1 — Yeast strains, expression plasmids and primers. (0.12 MB PDF) [file ppat.1001156.s001.pdf]

Table S1. Yeast Strains, Expression Plasmids and Primers

**yeast strains**	**Description**	**Source/reference**
BY4741	MATa his3Δ1 leu2Δ0 met15Δ0 ura3Δ0	Open Biosystems
met22Δ	met22::kanMX4 in BY4741 background	Open Biosystems
xrn1Δ	xrn1:: kanMX4 in BY4741 background	Open Biosystems
gcn4Δ	gcn4:: kanMX4 in BY4741 background	Open Biosystems
met22Δxrn1Δ	xrn1:: kanMX4, met22::hphNT1	This study
Gals-met22	Met22 promoter to GALS with kanMX4 in BY4741 background	This study
**Plasmids**	**Description**	**Source/reference**
pGADT7	LEU2 vector	Clontech
pGAD-His92/CUP1	His tagged CNV p92 under control of CUP1 promoter in LEU2 vector.	[1,2]
pGBK-His33/CUP1	His tagged CNV p33 under control of CUP1 promoter in HIS3 vector	[3]
pGBK-His33/DI72/CUP1	Coexpressing His tagged CNV p33 form ADH1 promoter and Di72 RNA from CUP1 promoter in HIS3 vector	[1,2]
pYC2-Di72	Di72 under GAL1 promoter in URA3 vector	[4]
pYC2-Di-AU-FP	DiAU under GAL1 promoter in URA3 vector	[5]
pYES2/NT	URA3 vector with GAL1 promoter	Invitrogen
pYES-Met22	6xHis N-tagged Met22 under contol of GAL1 promoter in URA3 vector	This study
pYES-MetA	His N-tagged Met22 with mutation in signature1 AA144-146	This study
pYES-MetB	His N-tagged Met22 with mutation in N-terminus AA25-30	This study
pYES-MetC	His N-tagged Met22 deletion of C-terminus at D311	This study
pYES-MetD	His N-tagged Met22 deletion of C-terminus at I292	This study
pYES-AHL*At*	His N-tagged AHL*At* under contol of GAL1 promoter in URA3 vector	This study
pFA6-hphNT1	PCR template for hphNT1 selection	[6]
pYM-N30	PCR template for GALS promoter and kanMX4 selection	[6]
pUC-CNV	CNV with T7 promoter	[7]
pTRV1	VIGS vector	[8]
pTRV2	VIGS vector	[8]
pTRV2-NbXRN4	405 bp NbXRN4 cDNA in VIGS vector	[9]
pTRV2-NbAHL	413 bp NbAHL cDNA in VIGS vector	This study
pTRV2-NbSAL1	347 bps of putative biphosphat nucleotidase from Nb	This study
pTRV2-NbFRY1	425 bps of nucleotidase similar to SAL2 from Nb	This study
pGD-CNV	CNV under control of 35S promoter	[1]
pGD-DiAU	DiAU under control of 35S promoter	[1]
pGD-ΔRI	Di-ΔRI under control of 35S promoter	[1]

**Primers**	**Description/Plasmid**
15 GTAATACGACTCACTATAGGGCATGTCGCTTGTTTGTTGG	T7/TBSV/RI/R
17 GTAATACGACTCACTATAGGAGAAACGGGAAGCTCGC	T7 Di72/RII/F
20 GGAAATTCTCCAGGATTTCTC	CNV/RI/F
22 GTAATACGACTCACTATAGGGCTGCATTTCTGCAATGTTCC	T7 probe CNV/R
157 GGGCTGCATTTCTGCAATGTTCC	TBSV1776/R
312 GCTGTCAGTCTAGTGGA	CNV/RIV/F
359 GTAATACGACTCACTATAGGAAATTCTCCAGGATTTC	T7/DI72/F
1660 TAATACGACTCACTATAGGAAGTTACAATTTATCCCCG	T7 probe RI CNV/R
2177 GGCGGGTACCCATGGCATTGGAAAGAGAATTATTG	pYES-Met22/F
2178 GGCGCTCGAGTTAGGCGTTTCTTGACTGAATGAC	pYES-Met22/R
2183 ATAGAATTCGATACAAGGAAAGGTACTATTCCG	pTRV2-NbXRN4/*F**
2187 ATATCTAGA TTACACCAGCCTGCCATGCAAAACGC	pTRV2-NbXRN4/*R**
2191 ATAGAATTC GATAATTCCCCTGTCACAATTGCTG	NbAHL/*F**
2192 ATATCTAGA TAATTTGGGCACCCGAGAACCCCAAG	NbAHL/*R**
2200 TAATACGACTCACTATAGG CTTTTATTTCGTTTAAAATTCCT	Met22/T7/R
2501 ATCCACGCCCTCCTACATC	hphNT1/confirmR
2581 TGTATGGTGCAGATGGAGAGAGCTCGGACACATACTGCGGCGTACGCTGCAGGTCGACGG	PromoterMet22/S1/F
2583 GTACAGCTTGAGTTGCAACCAATAATTCTCTTTCCAATGCCATCGATGAATTCTCTGTCG	Met22/S4/R
2584 GGCG GGATCC GTCGACCAAGGGGTTTTTAAGAGGTGA	pYES-MetA/F

2585 GGCGGAATTC GTCGACACTCCACTACTATTACCAAGA	pYES-MetB/F
2586 GGCG CTCGAG TTAATCTGTATGGATACCTCCAG	pYES-MetC/R
2587 GGCG CTCGAG TTAGATCTTTTCTTGGTAAGA	pYES-MetD/R
2588 GGCG GGATCC ATGGCGGTGGACTCCTTAGA	pYES-AHL*At/F*
2589 GGCG CTCGAG TCAGAGACTGGAAGATTCCC	pYES-AHL*At/R*
2590 GTAAAATATATGTTATTTAGGCGTTTCTTGACTGAATGACATCGATGAATTCGAGCTCG	Δmet22/S2/R
2591 CTTGGATGGGAAGTCCAAGA	Met22/500UTR/confirm
2754 TAATACGACTCACTATAGGAAGGGTCCGCTTCCACAAGTGA	T7 probe Di72/RIV/R
2755 AGTGTAAATCTGGCATAGCATAC	Di72/RIII/F
2733 TAATACGACTCACTATAGGGAAATCTCTCACCGTTTGGAATAG	T7 probe ITSrRNA/R
2734 TGGCAAGAGCATGAGAGCTTTTACTGGGC	ITSrRNA/F
2859 TAATACGACTCACTATAGGAACCAAATCATTCATGTTGCTCTC	Tubulin*/Nb1283/T7/R
2860 TAGTGTATGTGATATCCCACCAA	Tubulin*/Nb1080/F
2932 ATAGAATTC AGCCAGGCAAAGTATGGTGCTTTGTCC	NbFRY1*(BP135480) F
2933 ATATCTAGA CATAACAATTGAAGAGCTAAAGTAATA	NbFRY1*(BP135480) R
2935 ATAGAATTC TCAAGCCATTCATTCACAGCAGGACT	NbSall* (TA11598) F
2937 ATATCTAGA AAGGCTAGAGGAGTTCCAGCTAGCAT	NbSall* (TA11598) R
2940 AGGAACTCTCTCTTTCTCTTCCAAGAAA	AHL1* Nt N-region F
2941 TTCCTTAGAATGGACTTGTTCAAAGG	AHL1* Nt N-region R
KanB CTGCAGCGAGGAGCCGTAAT	KanMX/confirm/R

*DNA sequences are derived from blast searches using the sequences of *Arabidopis thaliana* genes in the *Solanaceae Genomics Resource* mRNAs (J.Craig Venter Institute database).

References

1. Jaag HM, Pogany J, Nagy PD (2010) A host Ca2+/Mn2+ ion pump is a factor in the emergence of viral RNA recombinants. Cell Host Microbe 7: 74-81.
2. Li Z, Barajas D, Panavas T, Herbst DA, Nagy PD (2008) Cdc34p Ubiquitin-Conjugating Enzyme Is a Component of the Tombusvirus Replicase Complex and Ubiquitinates p33 Replication Protein. J Virol 82: 6911-6926.
3. Jaag HM, Stork J, Nagy PD (2007) Host transcription factor Rpb11p affects tombusvirus replication and recombination via regulating the accumulation of viral replication proteins. Virology 368: 388-404.
4. Panavas T, Nagy PD (2003) Yeast as a model host to study replication and recombination of defective interfering RNA of Tomato bushy stunt virus. Virology 314: 315-325.
5. Serviene E, Jiang Y, Cheng CP, Baker J, Nagy PD (2006) Screening of the yeast yTHC collection identifies essential host factors affecting tombusvirus RNA recombination. J Virol 80: 1231-1241.
6. Janke C, Magiera MM, Rathfelder N, Taxis C, Reber S, et al. (2004) A versatile toolbox for PCR-based tagging of yeast genes: new fluorescent proteins, more markers and promoter substitution cassettes. Yeast 21: 947-962.
7. Cheng CP, Nagy PD (2003) Mechanism of RNA recombination in carmo- and tombusviruses: evidence for template switching by the RNA-dependent RNA polymerase in vitro. J Virol 77: 12033-12047.
8. Dinesh-Kumar SP, Anandalakshmi R, Marathe R, Schiff M, Liu Y (2003) Virus-induced gene silencing. Methods Mol Biol 236: 287-294.
9. Jaag HM, Nagy PD (2009) Silencing of Nicotiana benthamiana Xrn4p exoribonuclease promotes tombusvirus RNA accumulation and recombination. Virology 386: 344-352.
