# Supplementary material for: The Combined Effect of Environmental and Host Factors on the Emergence of Viral RNA Recombinants
Source: PLoS Pathog. 2010 Oct 21;6(10):e1001156. doi: 10.1371/journal.ppat.1001156 (PMC2958810; doi:10.1371/journal.ppat.1001156)
Supplement: Figure S1 — LiCl enhances the formation and accumulation of recRNAs and degRNAs in yeast. (A) Northern blot analysis of total RNA samples from yeast replicating TBSV DI-72 repRNA. BY4741 yeast cultures were treated with the shown concentration of LiCl. Samples were taken 24 hours after launching TBSV repRNA replication. Note that the repRNA was expressed from the GAL1 promoter only for the first 6 hours. (B) The percent of recRNA accumulation in comparison with the repRNA (100% in each sample) in yeast expressing DI-72 or DI-AU-FP repRNAs, respectively. (C) Northern blot analysis of total RNA samples from yeast replicating TBSV DI-AU-FP repRNA. See further details in panel A. (D) Schematic representation of the recRNA2 formed in yeast replicating DI-AU-FP. The replicase-driven template-switching event between two repRNAs is shown with an arrow. Note that the recombinants have variable sequences at the very 5′ end and at the junction sites, thus most of the recRNAs are different from one another. (0.64 MB PDF) [file ppat.1001156.s002.pdf]

Fig. S1

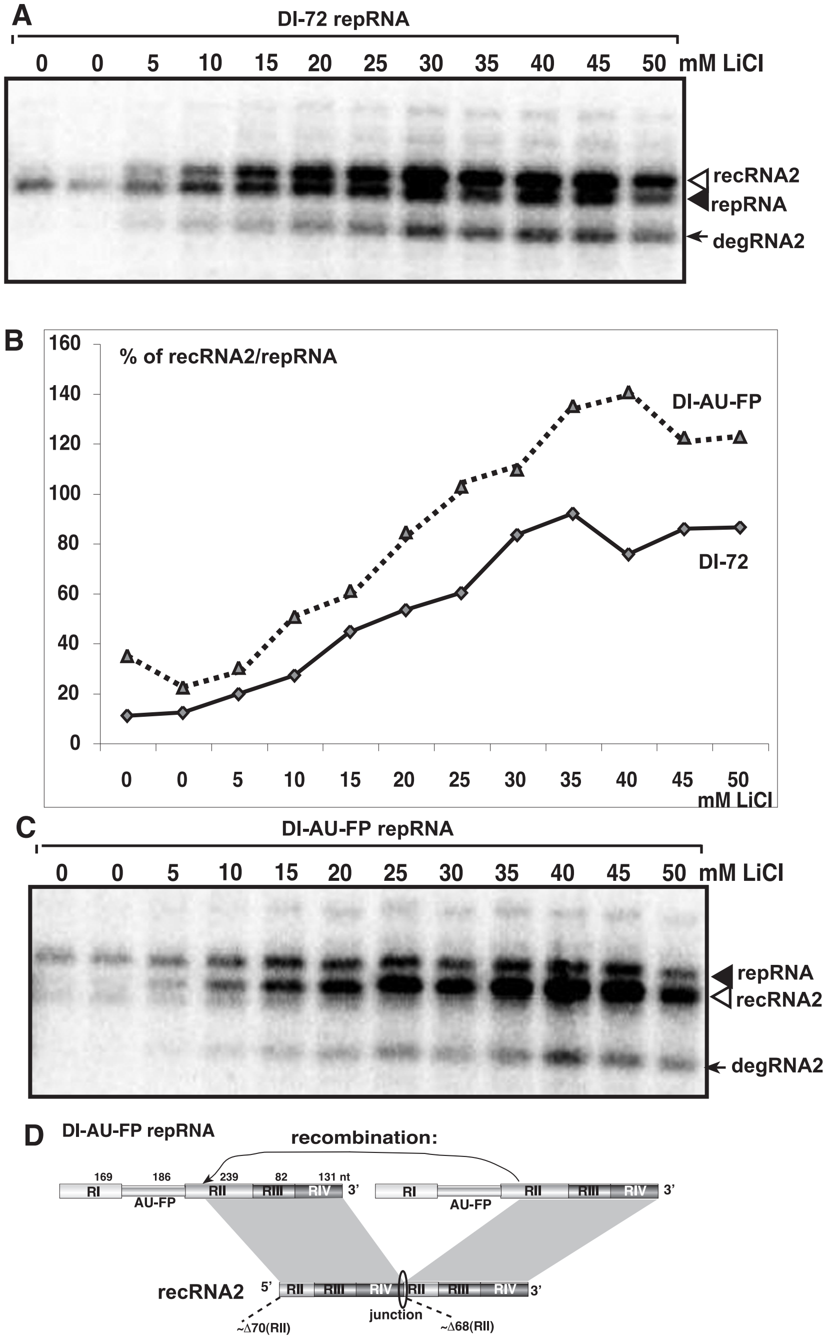
A
DI-72 repRNA
0 0 5 10 15 20 25 30 35 40 45 50 mM LiCl
recRNA2
repRNA
degRNA2
B
% of recRNA2/repRNA
160
140
120
100
80
60
40
20
0
DI-AU-FP
DI-72
0 0 5 10 15 20 25 30 35 40 45 50
mM LiCl
C
DI-AU-FP repRNA
0 0 5 10 15 20 25 30 35 40 45 50 mM LiCl
repRNA
recRNA2
degRNA2
D
DI-AU-FP repRNA
recombination:
169 186 239 82 131 nt
RI AU-FP RII RIII RIV 3'
RI AU-FP RII RIII RIV 3'
recRNA2 5' RII RIII RIV RII RIII RIV 3'
junction
~Δ70(RII)
~Δ68(RII)

Figure S1: LiCl enhances the formation and accumulation of recRNAs and degRNAs in yeast. (A) Northern blot analysis of total RNA samples from yeast replicating TBSV DI-72 repRNA. BY4741 yeast cultures were treated with the shown concentration of LiCl. Samples were taken 24 hours after launching TBSV repRNA replication. Note that the repRNA was expressed from the *GAL1* promoter only for the first 6 hours. (B) The percent of recRNA accumulation in comparison with the repRNA (100% in each sample) in yeast expressing DI-72 or DI-AU-FP repRNAs, respectively. (C) Northern blot analysis of total RNA samples from yeast replicating TBSV DI-AU-FP repRNA. See further details in panel A. (D) Schematic representation of the recRNA2 formed in yeast replicating DI-AU-FP. The replicase-driven template-switching event between two repRNAs is shown with an arrow. Note that the recombinants have variable sequences at the very 5' end and at the junction sites, thus most of the recRNAs are different from one another.
