# Supplementary material for: The Combined Effect of Environmental and Host Factors on the Emergence of Viral RNA Recombinants
Source: PLoS Pathog. 2010 Oct 21;6(10):e1001156. doi: 10.1371/journal.ppat.1001156 (PMC2958810; doi:10.1371/journal.ppat.1001156)
Supplement: Figure S2 — Increased level of accumulation of the nondegraded ITS1 region of pre-ribosomal RNA after LiCl treatment. The activity of cellular 5′-3′ exoribonucleases, such as Xrn1p (cytosolic) and Rat1p (nucleus), was inhibited by the shown amount of LiCl as described. As expected, LiCl treatment increased the accumulation of pre-ribosomal RNA carrying the ITS1 region by up to 7-fold, which is indicative of reduced level of Xrn1p and Rat1p nuclease activities in yeast cells. (0.22 MB PDF) [file ppat.1001156.s003.pdf]

Fig. S2

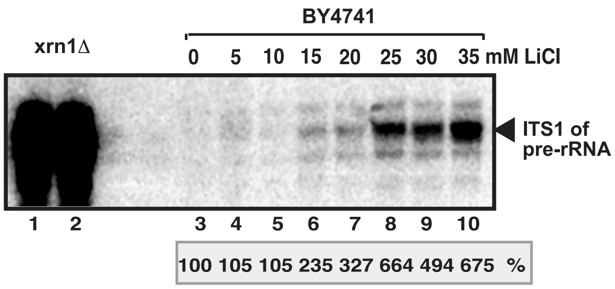

BY4741
xrn1Δ
0 5 10 15 20 25 30 35 mM LiCl
ITS1 of
pre-rRNA
1 2 3 4 5 6 7 8 9 10
100 105 105 235 327 664 494 675 %

Figure S2: Increased level of accumulation of the nondegraded ITS1 region of pre-ribosomal RNA after LiCl treatment. The activity of cellular 5'-3' exoribonucleases, such as Xrn1p (cytosolic) and Rat1p (nucleus), was inhibited by the shown amount of LiCl as described. As expected, LiCl treatment increased the accumulation of pre-ribosomal RNA carrying the ITS1 region by up to 7-fold, which is indicative of reduced level of Xrn1p and Rat1p nuclease activities in yeast cells.
